# PD-1抑制剂治疗慢性活动性EB病毒感染6例报告并文献复习

**DOI:** 10.3760/cma.j.issn.0253-2727.2023.02.016

**Published:** 2023-02

**Authors:** 佳颖 吴, 斌 徐, 晓健 朱, 茜 明, 辉 罗, 霞 毛, 佳 顾, 剑峰 周, 毅 肖

**Affiliations:** 华中科技大学同济医学院附属同济医院血液内科，武汉 430030 Department of Hematology, Tongji Hospital, Tongji Medical College, Huazhong University of Science and Technology, Wuhan 430030, China

慢性活动性EB病毒感染（CAEBV）是一种相对罕见但是预后较差的T/NK细胞增殖性疾病，通常以持续或反复发作的传染性单核细胞增多症样症状为主要临床表现，同时伴有EB病毒（EBV）DNA水平升高[1]–[2]。一般而言，传统的抗病毒治疗以及常规化疗的治疗效果欠佳，若是不能得到及时有效的治疗，CAEBV可能发展为EBV相关的噬血细胞性淋巴组织细胞增多症（EBV-HLH）、多器官功能障碍综合征、淋巴瘤等疾病[3]–[4]。异基因造血干细胞移植（allo-HSCT）是当前可以治愈CAEBV的唯一方法，但是处于疾病活动状态时移植结局往往不良，因此，如何有效地控制急性期症状以便于更好桥接后续治疗显得尤为重要[5]–[6]。T细胞功能障碍是CAEBV重要的始动因素，转录组测序发现程序性死亡受体1（PD-1）及其配体（PD-L1）信号通路异常，提示免疫检查点治疗可能是治疗CAEBV的一种创新型策略[7]–[8]。近来，Liu等[9]的研究发现PD-1抑制剂Nivolumab可以通过恢复体内有缺陷的抗EBV作用为复发、难治性EBV-HLH患者提供潜在的治愈能力。CAEBV通常被认为是EBV-HLH发病的可能根源，然而，关于PD-1抑制剂在CAEBV的应用，目前尚无明确的数据报道。我们回顾性收集了6例接受PD-1抑制剂治疗的CAEBV患者临床资料，初步探索PD-1抑制剂在CAEBV中的应用前景。

## 病例与方法

1. 病例来源：回顾性收集和分析2017年1月1日至2021年12月31日在华中科技大学同济医学院附属同济医院血液内科接受PD-1抑制剂治疗的CAEBV患者的临床资料，数据收集前通过华中科技大学同济医学院伦理学委员会批准，并且获得患者及其家属的知情同意。

2. 治疗方案：每3～4周静脉注射1次PD-1抑制剂信迪利单抗，常规剂量200 mg，为1个疗程，至少持续3个疗程。发生≥3级的药物相关不良反应时停止使用。若患者临床症状缓解、血浆EBV-DNA拷贝数转阴，剂量调整为每3个月100 mg，维持治疗至少1年。

3. 实验室检查：采用EBV分类PCR（EBV-Sorting PCR）技术确定感染的靶细胞类型，多参数流式细胞术检测CD107a鉴定NK细胞功能。目前CAEBV的疗效评估尚缺乏统一的标准。每个疗程输注前，由专业医师对临床疗效和药物不良反应进行综合评估，包括患者的临床症状和体征、血浆和外周血单个核细胞（PBMC）EBV-DNA拷贝水平、血细胞计数、丙氨酸转氨酶（ALT）、天冬氨酸转氨酶（AST）、总胆红素（TBIL）和肌酐（Cr）水平。

4. 随访：随访截至2021年12月31日。无进展生存（PFS）期定义为治疗开始到疾病出现进展、任何原因死亡或末次随访的时间。

## 结果

1. 患者基线特征：共6例CAEBV患者被纳入分析，其中男4例，女2例，中位年龄33（13～42）岁。既往抗病毒治疗、激素或化疗复发难治者4例，初治者2例。首诊临床症状和体征主要包括发热，肝、脾肿大，肝功能异常，淋巴结肿大和血细胞减少等。除例3外，其余患者治疗前PBMC EBV-DNA拷贝数均超过1×10^4^拷贝数/ml；治疗前2例患者血浆EBV-DNA拷贝数阴性。EBV-Sorting PCR检测显示所有患者均以感染NK细胞为主，5例NK细胞杀伤活性下降。例2的骨髓细胞学检查示噬血现象，达到HLH的诊断标准，考虑该患者合并EBV-HLH。更多患者基本信息详见表1。

**表1:** 6例接受信迪利单抗治疗的慢性活动性EB病毒感染患者基线特征、治疗情况及临床疗效

病例	年龄（岁）	性别	主要症状、体征	是否复发难治	治疗周期	总剂量（mg）	外周血单个核细胞EBV-DNA（拷贝数/ml）		血浆EBV-DNA（拷贝数/ml）		不良反应	临床结局
							治疗前	治疗后	治疗前	治疗后		
1	13	女	发热、肝功能异常、肝脾肿大	复发难治	6	1 200	7.45×10^6^	4.42×10^6^	1.27×10^4^	3.67×10^3^	无	疾病稳定
2	42	男	发热、肝脾肿大、血细胞减少、肝功能异常	初治	5	1 000	3.96×10^5^	2.61×10^6^	5.09×10^2^	阴性	胰腺炎	拒绝继续治疗，失访
3	15	男	淋巴结肿大	复发难治	9	1 000	9.15×10^2^	9.56×10^3^	3.48×10^3^	2.06×10^3^	肝功能异常	疾病稳定
4	42	男	鼻塞、淋巴结肿大	初治	8	1 300	1.84×10^4^	2.36×10^3^	阴性	阴性	无	进展为NK/T细胞淋巴瘤
5	30	男	口腔溃疡、嘴唇及面部肌肉肿胀	复发难治	18	2 600	3.28×10^6^	阴性	阴性	阴性	无	疾病稳定
6	36	女	发热、淋巴结肿大	复发难治	14	1 400	1.02×10^5^	阴性	5.24×10^3^	阴性	无	疾病稳定

2. 临床疗效：见表1。所有患者接受规律的信迪利单抗治疗，中位治疗8.5（5～18）个疗程，中位输注剂量为1 250（1 000～2 600）mg。治疗后，所有患者的急性活动期临床症状均得到明显缓解，发热者体温均下降至正常范围；淋巴结肿大者较前明显缩小（例4）或恢复正常（例3、6）；肝脾肿大者较前缩小；例2血常规逐渐恢复正常，未见噬血现象加重。此外，原血浆EBV-DNA拷贝数阳性的患者均有一定程度的下降，其中例2、6完全转阴，原阴性的患者则可以继续保持。而对于PBMC EBV-DNA拷贝数而言，例1、4治疗后有所降低，例5、6转阴，但是例2、3出现了轻微升高。中位随访38.2（3.5～57.0）个月，中位PFS未达到，预估3年PFS率为75.0％（95％ *CI* 12.8％～96.1％）。其中，例2因药物相关不良事件拒绝继续治疗，失访；例4在完成8个疗程的治疗后，疾病保持稳定状态，但是随访20个月后，患者进展为NK/T细胞淋巴瘤，后续进行规律化疗；其余4例患者（均为既往复发难治）保持疾病稳定状态，未见明显急性活动期临床症状。

例5在初诊时存在口腔溃疡，嘴唇、面部肌肉肿胀和PBMC EBV-DNA拷贝数升高，治疗后临床症状完全缓解，血浆和PBMC中均检测不到EBV-DNA拷贝。此外，经过8个周期的输注后，复查PET/CT发现，嘴唇和面部肌肉的肿胀明显减轻，左上肢和胸肌处原有的高代谢病变消失，右上肢和颈部的高代谢病变也明显减少，原骨髓弥漫性高代谢明显下降，虽然颈部双侧淋巴结仍有肿大，但是代谢正常。

3. 不良反应：整个治疗过程中，未观察到严重危及生命的药物相关不良事件，仅例2出现信迪利单抗使用相关的胰淀粉酶和脂肪酶升高，结合影像学诊断考虑为胰腺炎，参照常见不良事件评价标准5.0，评估为2级，该患者拒绝进一步治疗，后失访。治疗后所有患者肝肾功能指标变化见图1，TBIL、Cr在正常范围内波动，例3出现短暂性ALT与AST轻度升高，例1、2治疗前存在可能因疾病活动所致的肝酶升高，治疗后逐渐下降。

**图1:**
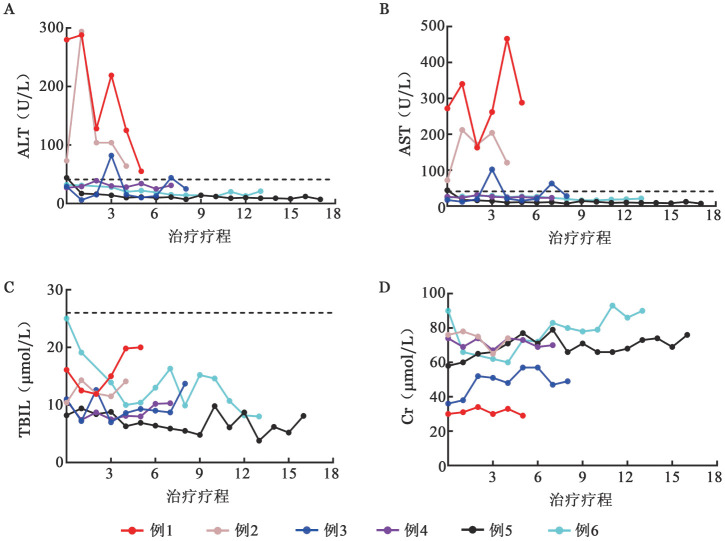
6例慢性活动性EB病毒感染患者信迪利单抗治疗后肝、肾功能指标变化 A ALT；B AST；C TBIL；D Cr **注** ALT：丙氨酸转氨酶；AST：天冬氨酸转氨酶；TBIL：总胆红素；Cr：肌酐

## 讨论

CAEBV是一种主要感染T细胞和NK细胞的EBV相关的淋巴细胞增殖性疾病，具有侵袭性和致命性[1]–[2]。在CAEBV治疗方案的选择上，日本学者提出了“三步疗法”，尽管激素、常规化疗以及其他治疗方法（如EBV特异性细胞毒性T淋巴细胞、组蛋白去乙酰化酶抑制剂等）可以在一定程度上延缓疾病的自然进程，但是若没有接受allo-HSCT，患者最终会因疾病进展死亡[3],[5],[10]。因此，探索除移植外新型有效的治疗策略是当前CAEBV面临的重要挑战。一项单中心、回顾性研究显示，JAK1/2抑制剂芦可替尼虽然在控制CAEBV患者急性期发热症状方面效果显著，但在降低EBV-DNA拷贝数方面并不理想[11]。近年来，以PD-1/PD-L1阻断为代表的免疫检查点疗法开启了恶性淋巴瘤免疫治疗的新时代[12]–[13]。有研究显示该疗法对EBV-HLH亦有潜在治愈力[9]。因此，我们回顾性收集和分析既往在我院接受信迪利单抗治疗的CAEBV患者的临床资料，初步结果提示信迪利单抗可以有效降低血浆EBV-DNA拷贝水平，控制CAEBV患者疾病活动状态。

本研究一共纳入6例CAEBV患者，其中4例为复发难治，在接受信迪利单抗治疗后，急性期临床症状均得到明显缓解。血浆EBV-DNA拷贝水平作为许多EBV相关恶性疾病的重要评价指标，临床应用较为广泛[14]–[15]。通常情况下，正常成人或潜伏期感染患者的血浆EBV-DNA拷贝往往低于检测下限或者呈现低水平。本研究中，患者在接受信迪利单抗治疗后，血浆EBV-DNA拷贝数均有一定程度的下降，其中2例患者完全转阴。然而，对于PBMC EBV-DNA而言，虽然多数患者呈减低趋势，但是有2例患者轻微升高，这与Liu等[9]的报道不一致。提示PD-1/PD-L1阻断疗法可能是控制CAEBV患者急性活动期症状的一种有希望的治疗策略，但疗效有限，不能治疗潜伏期感染，无法达到根治疾病的目的。这可能与CAEBV发病机制涉及到基因层面的异常相关，既往研究发现，T细胞或NK细胞的细胞毒活性受损通常是由于DDX3X或其他基因突变缺失所致，而基因突变一般不会被PD-1抑制剂等表观遗传因素所逆转[16]。尽管如此，急性活动期症状的有效缓解仍可为后续的桥接治疗如allo-HSCT提供机会，改善临床预后。

EBV感染与结外NK/T细胞淋巴瘤（ENTKL）的发生密切相关，全基因组表达谱分析结果显示，相较于正常对照组织，ENTKL PD-L1 mRNA表达水平上调，并且与EB病毒潜伏膜蛋白1（LMP1）呈正相关[17]–[18]。Xu等[19]联合PD-1抑制剂、西达本胺和局部放疗挽救治疗1例复发进展的ENKTL患者，获完全缓解。因此，PD1/PD-L1信号通路在多种EBV相关性疾病的发生、发展中占有十分重要的地位。然而，目前对于该信号通路在CAEBV中作用的认识尚不完整。既往研究发现，T淋巴细胞所介导的免疫反应在EBV感染性疾病的演变过程中起关键作用，CAEBV患者EBV重新激活进入裂解期可能与潜在的免疫缺陷有关。与健康携带者相比，CAEBV或传染性单核细胞增多症患者外周血中TCRβ重排多样性明显下降，CAEBV患者体内EBV特异性CD8^+^ T淋巴细胞数量下降且功能异常[20]–[21]。由此可见，T淋巴细胞功能障碍是CAEBV的重要发病机制之一。Liu等[9]的报道显示PD-1抑制剂可以恢复EBV-HLH患者体内CD8^+^ T淋巴细胞的抗EBV能力。我们的结果提示，PD-1抑制剂可以控制EBV急性裂解，猜想可能通过恢复T淋巴细胞数量及功能实现，但由于缺乏治疗前后T淋巴细胞亚群及功能对比结果，具体机制仍需进一步验证和探索。

总体而言，采用PD-1抑制剂治疗CAEBV的初步研究结果表明PD-1抑制剂可以有效降低血浆EBV-DNA拷贝水平，控制CAEBV患者的急性活动期临床症状，为后续allo-HSCT或其他治疗提供机会，改善患者的临床预后。但是本研究的病例数较少，此外，目前只进行了临床疗效和不良反应的观察，并未对其机制深入探索，后续还需进一步研究。
